# Differential Healing Patterns of Mucosal Seal on Zirconia and Titanium Implant

**DOI:** 10.3389/fphys.2019.00796

**Published:** 2019-07-03

**Authors:** Dong-Joon Lee, Joon-Sang Ryu, Masaki Shimono, Keun-Woo Lee, Jong-Min Lee, Han-Sung Jung

**Affiliations:** ^1^Division in Anatomy and Developmental Biology, Department of Oral Biology, Oral Science Research Center, BK21 PLUS Project, Yonsei University College of Dentistry, Seoul, South Korea; ^2^Department of Prosthodontics, Oral Science Research Center, Yonsei University College of Dentistry, Seoul, South Korea; ^3^Department of Pathology, Tokyo Dental College, Tokyo, Japan; ^4^Key Laboratory of Oral Medicine, Guangzhou Institute of Oral Disease, Stomatology Hospital of Guangzhou Medical University, Guangzhou, China

**Keywords:** zirconia implant, laminin-332, biological width, peri-implant epithelium elongation, mucosal seal

## Abstract

Zirconia implants have become an alternative to titanium implants due to several advantages. The zirconia implant is relatively esthetic and highly resistant to bacteria. While biomaterial studies for zirconia implants have considerably accumulated, *in vivo* studies have not yet progressed. In the present study, the functional and biological properties of zirconia implants were analyzed thorough *in vitro* and *in vivo* studies. The proliferation properties of periodontal cells on the discs of machined surface titanium, hydroxyapatite coated titanium and zirconia were analyzed, and zirconia was shown to be favorable. In addition, small implant fixtures that can be applied to the jawbone of mice were manufactured and transplanted to C57BL/6 mice. The adhesion molecules expression patterns in peri-implant mucosa suggest a stronger mucosal seal and more adequate prevention of peri-implant epithelium (PIE) elongation in the zirconia implant when compared with other conventional materials. Differential laminin-332 expression in peri-implant mucosa of zirconia implants seems to regulate the PIE elongation. In conclusion, zirconia was found to be promising and advantageous with regards to the mucosal seal. And biological width (BW) of peri-implant mucosa is more desirable in zirconia implants compared to conventional titanium implants.

## Introduction

Since the introduction of dental implants for clinical applications, titanium has been considered the standard material for the treatment of edentulous jaws (Nishihara et al., 2018). The use of dental implants to rehabilitate the loss of teeth has increased in the last 30 years (Jenny et al., 2016). Currently, dental implants are considered a viable treatment option for replacing missing teeth that have been either extracted or have been ejected due to caries or periodontal disease in clinic (Hong and Oh, 2017). Clinical studies have validated the long-term success of titanium dental implants for the treatment of missing teeth (Branemark et al., 1977; Adell et al., 1981). Although titanium has been in use for more than 40 years, a number of criticisms regarding its clinical application has been raised (Branemark et al., 1984; Branemark et al., 2001).

In order to overcome these criticisms, the modification of the implant surface has been studied and applied to improve the biological surface properties favoring osseointegration (Smeets et al., 2016) or bacterial resistance (Damiati et al., 2018; Orapiriyakul et al., 2018). Several surface modifications have been introduced, among which hydroxyapatite (HA) coating is estimated to have achieved significant improvements in osseointegration formation time and strength (Buser et al., 1991; Jemat et al., 2015). Although many surface modifications have been developed, including HA coating, new materials have been introduced due to the inherent limitation of the material, titanium.

Zirconia implants have become an alternative to titanium implants due to several advantages (Ozkurt and Kazazoglu, 2011). Titanium implants lead to a faint gray background of the thin peri-implant mucosa or recession (Jung et al., 2008). This discoloration has become an esthetic disadvantage. In other words, the zirconia implant has an aesthetic advantage due to being of a similar shade as the tooth root. In addition, various reports concluded that exposure to titanium could lead to hypersensitivity (Lalor et al., 1991; Gawkrodger, 2005; Hosoki et al., 2009). Zirconia implants are also highly resistant to bacteria. In some studies, surface treatment results in zirconia surfaces being more resistant to bacterial adhesion than titanium surfaces (Buczynski et al., 2003; Al-Radha et al., 2012; de Oliveira et al., 2012). A study suggested titanium-zirconium (TiZr) alloy as a new material due to a high affinity with human gingival fibroblasts (Gomez-Florit et al., 2014). A commercially available zirconia implant system has been launched and is in long-term clinical trials in South Korea.

The causes of periodontal and peri-implant diseases are various and include environmental factors such as smoking, local factors such as oral bacterial flora, and systemic factors such as nutritional status, hematologic disorder and hormonal abnormality (Reynolds, 2014; Holtfreter et al., 2015; Lee et al., 2018). The major and most common cause of implant failure is peri-implantitis, and, for structural reasons, the mucosal seal between the implant and adjacent mucosa is closely related to the initiation of peri-implantitis (Ramanauskaite and Juodzbalys, 2016; Schwarz et al., 2018). The peri-implant epithelium (PIE) as a component of the mucosal seal acts as a primary physical barrier of the peri-implant mucosa. Laminin-332, also known as laminin-5, has been reported as the key molecule of mucosal wound healing and mucosal seal formation in the junctional epithelium (JE) in the natural gingiva and PIE in numerous studies (Atsuta et al., 2005a; Larjava et al., 2011; Zhang et al., 2018).

Biological width (BW) is defined as the dimension of the soft tissue, which is attached to the portion of the tooth coronal to the crest of the alveolar bone (Nugala et al., 2012). The BW consists of JE and underlying connective tissue (CT). The BW of natural tooth is reported as 2.04 mm (JE: 0.97 mm, CT: 1.07 mm) (Gargiulo et al., 1961), which is considered orthodoxy. The BW is essential for the preservation of periodontal health and removal of irritation that might damage the periodontium (Nugala et al., 2012).

The aims of the present study were (1) to determine the characteristics of periodontal cells on zirconia, hydroxyapatite coated titanium and machined surface titanium, (2) to evaluate the expression of mucosal adhesion molecules including laminin-332 around zirconia and other implants, (3) to describe the ratio of epithelium and connective tissue within the soft tissue attachment on the zirconia and other implants and (4) to provide evidence for determining which material is more advantageous for clinical use.

In the present study, small implant fixtures that can be applied to the jawbone of mice were manufactured. There were differences in the composition of the biological width according to the materials. The expression of the adhesion molecule forming the mucosal seal was also slightly different. In addition, the evidence suggested stronger mucosal seal formation with zirconia fixtures than with other materials. PIE elongation (or migration) prevention was also observed in the zirconia implant. This phenomenon appears to be related to laminin-332. Zirconia was advantageous with regards to the mucosal seal; on the bone healing side, Hydroxyapatite (HA)-coated titanium was favorable.

## Materials and Methods

All the animal experiments were approved by the Yonsei University Health System Institutional Animal Care and Use Committee (YUHS-IACUC) in accordance with the Guide for the Care and Use of Laboratory Animals (National Research Council, United States). The animal study plan for these experiments (2016-0342) was reviewed and approved by the committee on January 28, 2017. All the experiments were performed in accordance with the guidelines of the committee.

### Cell Culture on Titanium and Zirconia Discs

Human periodontal ligament fibroblasts (HPLFs, #2630) were purchased from ScienCell Research Laboratories (Carlsbad, CA, United States) and cultured with a fibroblast medium (ScienCell, United States, #2301). The immortalized human cementoblasts (ihCEMs) were obtained from professor Takata’s laboratory (Hiroshima University, Japan). ihCEMs were immortalized by transfection with the telomerase catalytic subunit, the hTERT genes (Kitagawa et al., 2006). They were cultured with Minimum Essential Medium-alpha (MEM-α, Gibco, Life Technologies, United States, 12571-063) with 10% FBS and 1% P/S solution. The human gingival fibroblast cells (HGF-1) were purchased from ATCC (Manassas, VA, United States) and cultured with Dulbecco’s Minimum Eagle’s Medium (DMEM, Gibco, Life Technologies, United States, 11995-065) with 10% fetal bovine serum (FBS, Gibco, Life Technologies, United States, 12484-020), and 1% penicillin/streptomycin (P/S) solution. All the cells were incubated under 37°C and 5% CO_2_ conditions.

The titanium discs, HA-coated discs of 10.0 mm diameter were purchased from Shinheung, Co. (Seoul, South Korea). The zirconia discs were made with (Y,Nb)-TZP in 10.0 mm diameter by the Dental laboratory (Yonsei University College of Dentistry, South Korea) (Supplementary Figure 1A). The three types of periodontal cells were seeded on each type of five discs in a 24-well cell culture plate (SPL Life Sciences, South Korea, 30024) or on five wells of the plate. The well diameter of the 24-well dish was 15.6 mm and the diameter of discs was 10.0 mm. According to the area ratio, 20,000 cells were seeded on each disc and 50,000 cells were seeded on the control dishes. After 6 h of allowing the cells to settle on the discs, 900 μL culture medium was carefully added to each disc-containing well. The medium was changed after every cell proliferation assay.

### Cell Proliferation Assay

A Cell Counting Kit-8 (Dojindo Molecular Technologies, Inc., Kumamoto, Japan) was used for the cell proliferation assay. All the medium was removed and 500 μL fresh medium was added to each well including an empty well for a medium background measurement (negative control). Then, 50 μL water-soluble tetrazolium salt (WST-8) reagent was added to each well including the negative control. After 1 h of CO_2_ incubation, all the media were transferred to a 96-well cell culture plate (SPL Life Sciences, South Korea, 30096). The absorbance was then measured three times at 450 nm with a microplate reader (Benchmark Plus, Bio-Rad, CA, United States). The number of cells was measured as the survival rate with the equation below:

survival rate = Asample-AblankAcontrol-Ablank

(A_control_: negative control; A_blank_: blank well).

The number of cells was measured at 24, 72, and 120 h after seeding. The number of cells on the control dish measured at 24 h after seeding was defined as 1.0 for each assay. And the cell numbers on the discs were calculated as a ratio to the 24 h cell number of the control dish. The cell proliferation assay was repeated three times.

### Animals

Female C57BL/6 mice (Narabiotech Co., Pyeongtaek, South Korea) were housed in a temperature-controlled room (22°C) under artificial illumination with a 12 h light/dark cycle and 55% relative humidity. The mice were provided food and water ad libitum. All the operational procedures were performed under deep anesthesia. Fifteen female mice were randomly divided into three groups (*n* = 5 per group).

### Implant Fixtures and Transplantation

Three types of implants were designed to be small enough to be applied to the oral space of the mice: the machined surface titanium (Ti), HA-coated titanium (HA) and machined surface zirconia (Supplementary Figures 1B,D). The titanium implants were generated from pure titanium and shaved to produce threads at the apical side with a driver slot on the head. The thread of the implants was 1.42 mm in length and 1.00 mm in diameter. For the HA-coated titanium implant, the thread surface was coated with HA at an average thickness of 1.86 μm (Daechang Metal, Co., South Korea). The zirconia implants were generated from (Y,NB)-TZP zirconia blocks in the same gauge as the titanium implants (Kaiser Precision, Co., South Korea).

The upper first molars of 6-week-old mice were extracted while under deep anesthesia and allowed to heal for 6 weeks. Under deep anesthesia, a pilot hole was made using a portable, low-speed engine with a 0.75 mm drill tip designed for HA implants. Subsequently, the fixture was transplanted into the hole using a driver (Supplementary Figure 1E). The cusps of opposing tooth (mandibular first molar) were removed to avoid the fall out of fixtures due to occlusion. The implant-transplanted mice were housed for 8 weeks in the animal room, as described previously, for healing. The mice were subsequently euthanized with CO_2_.

### Immunohistochemistry (IHC)

The tissues were excised and immersed in 4% paraformaldehyde (PFA). After fixation, the tissues were decalcified in 10% sodium citrate and 22.5% formic acid for 6 weeks at 4°C. Staining was performed on 6 μm paraffin-embedded sagittal (mesio-distally) sections. After deparaffinization, the slides were incubated with Proteinase K (10 μg/mL, AM2546, Thermo Fisher Scientific, United States) for 20 min at 37°C. Subsequently, the slides were incubated with antibodies against collagen IV (1:500 dilution, ab6586, Abcam, United Kingdom), fibronectin (1:400 dilution, ab2413, Abcam, United Kingdom), plakophilin (1:100 dilution, ab230855, Abcam, United Kingdom), or laminin-5 (1:200 dilution, ab14509, Abcam, United Kingdom) at 4°C overnight. The specimens were sequentially incubated with secondary antibodies and streptavidin peroxidase. The results were visualized following staining with a diaminobenzidine (DAB) reagent kit (Invitrogen, United States). The sections were counterstained with Mayer’s hematoxylin. All the specimens were observed using a stereomicroscope (MD5500D; Leica, camera: DFC495; Leica, Lens: HCX PL APO 409; Leica).

### Measurements on Histological Images

The bone area and biological widths were measured using the ImageJ shareware software (ver. 1.38e, NIH, United States). For bone area measurements, two slices with fixture diameters greater than 600 μm were selected from each dissected maxilla sample. The ROI was set from the top of the thread part of the fixture to the third thread crest and 150 μm from the surface of the fixture (Figure 2d–f, dotted lines). Empty areas such as blood vessels were excluded from the ROI, and the area occupied by the bone in the ROI was measured. Measurements were made on both the mesial and distal sides of two selected slices of each harvested samples (four ROIs per sample). The numbers of harvested Ti and zirconia samples were four each. And five samples of HA were harvested. For biological widths measurement, the same slices used as for bone area measurement. Eight slices of four mice were used. biological widths were measured from totally 16 mesial and distal sites from 8 slices in Ti and zirconia samples. Twenty sites from 10 slices of 5 mice samples were measured for HA. For measurement of natural tooth biological widths, opposing teeth of HA implanted five mice were used. Biological widths of 10 distal sites of first molars and 10 mesial sites of second molars were measured.

### Statistical Analysis

All the numeric data including cell proliferation, bone area and biological width compositions were expressed as the mean ± standard deviation (SD). The statistical analyses were performed by one-way analysis of variance (ANOVA) followed by the Student’s *t*-test. For all the analyses, *p* < 0.05 was considered significant.

## Results

### Proliferation of Periodontal Cells on Titanium and Zirconia

**FIGURE 1:**
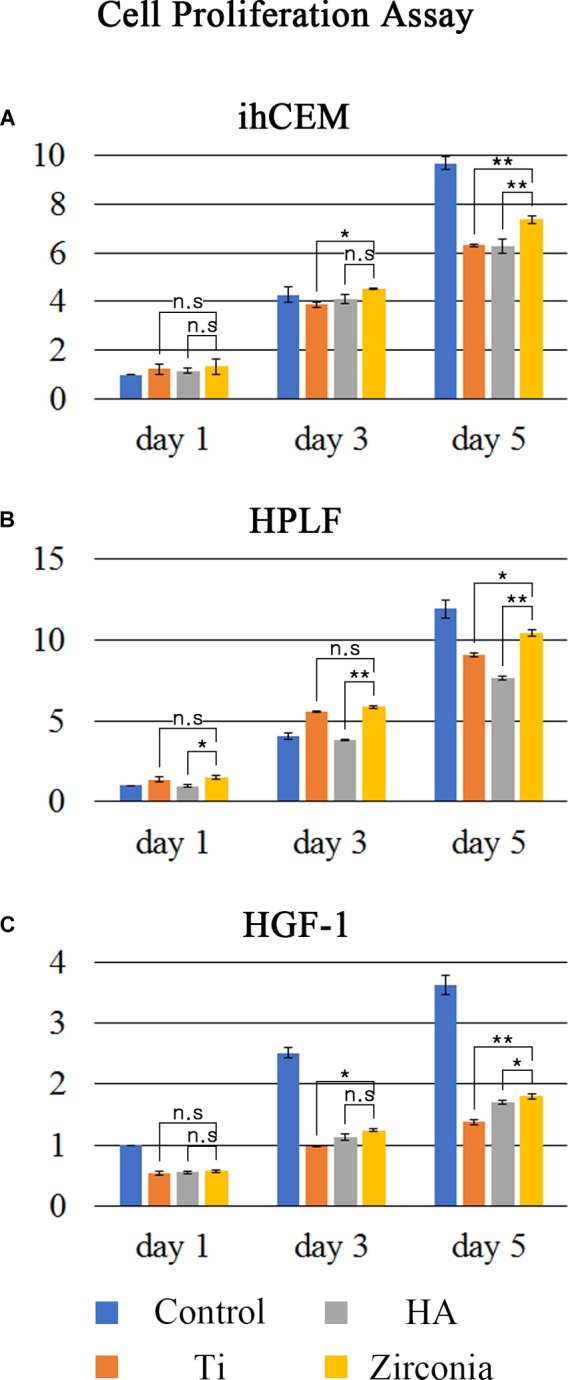
Cell proliferation assay. Cell proliferation ratios were measured using the WST-8 assay. **(A)** ihCEM, **(B)** HPLF, and **(C)** HGF-1 cells were cultured on normal cell culture dishes (Control), titanium discs (Ti), HA-coated titanium discs (HA), or zirconia discs (Zirconia). The number of discs used in the measurement was five per group. The proliferation data of the cells were normalized with Day 1 control. The cell proliferation ratio was higher on the zirconia discs than on the other two discs. ^∗^*p* < 0.05, ^∗∗^*p* < 0.01.

The ihCEMs, HPLFs and HGF-1 cells were cultured on a conventional 24-well cell culture dish (Control), machined surface titanium discs (Ti), HA-coated titanium discs (HA) or zirconia discs (Figure 1). All the numbers of cells in control dish and discs increased significantly with time (*p* < 0.01 for ihCEM and HPLF; *p* < 0.05 for HGF-1), and proliferation rates of all three types of cells on control dishes were higher than those of cells on discs at day 5.

The ihCEMs proliferated more on the zirconia discs than on the Ti or HA discs at 3–5 days (Figure 1A). The proliferation rates of ihCEMs at day1 were not significantly different. However, the relative cell number (fold change to control dish at day 1) on the zirconia discs (4.507 ± 0.028; mean ± SD) was higher than on Ti discs (3.870 ± 0.114), but not than HA discs (4.084 ± 0.177) at day 3. Zzirconia vs. Ti; *p* = 0.032, zirconia vs. HA; *p* = 0.064 were considered significant. At day 5, the zirconia discs (7.360 ± 0.145) had a higher cell number than Ti discs (6.304 ± 0.056) or HA discs (6.272 ± 0.288). These were statistically significant (zirconia vs. Ti; *p* = 0.006, zirconia vs. HA; *p* = 0.010). There was no significant difference between Ti discs and HA discs.

The HPLFs on zirconia showed similar patterns to ihCEMs. Among the three types of discs, zirconia had the highest proliferation rate (Figure 1B). Up to day 1, the relative number of cells on the zirconia discs (1.488 ± 0.108) was higher than that of HA discs (0.971 ± 0.101) (*p* = 0.046). No difference was observed between zirconia and Ti discs (0.369 ± 0.181, p = 0.234). The zirconia discs (5.864 ± 0.088) had a higher relative number of cells than the HA discs (3.827 ± 0.025), but not higher than Ti discs (5.589 ± 0.034) at day 3. Zirconia vs. Ti; *p* = 0.160, zirconia vs. HA; *p* = 0.009 was considered statistically significant. At day 5, the zirconia discs relative number of cells (10.420 ± 0.194) was higher than on Ti discs (9.070 ± 0.102) or HA discs (7.611 ± 0.128). These were statistically significant (zirconia vs. Ti; *p* = 0.025, zirconia vs. HA; *p* = 0.008).

HGF-1 cells proliferated twice more on the control dish than on the experimental discs (Figure 1C). Among the three discs, HGF-1 cells proliferated best on zirconia discs at days 3 and 5. The relative cell number on the zirconia discs (1.245 ± 0.027) was higher than on Ti discs (0.982 ± 0.016), but not higher than on HA discs (1.128 ± 0.056) at day 3. This was statistically significant (zirconia vs. Ti; *p* = 0.037, zirconia vs. HA; *p* = 0.051). At day 5, the relative cell number on the zirconia discs (1.801 ± 0.044) was higher than on Ti discs (1.376 ± 0.043) or HA discs (1.670 ± 0.033). These were statistically significant (zirconia vs. Ti; *p* = 0.018, zirconia vs. HA; *p* = 0.048). There was no difference among the three discs at day 1. Unlike the other two cells, the proliferation of HGF-1 on the discs was significantly lower than the proliferation on the control dish.

### Bone Healing After the Fixture Transplantation

To investigate the bone healing and osseointegration of the implant, three different types of fixtures were used in this study. HA fixture surface was rugged and considered to be a relative high surface area, while Ti and zirconia fixture had flat machined surfaces (Supplementary Figure 1C).

**FIGURE 2:**
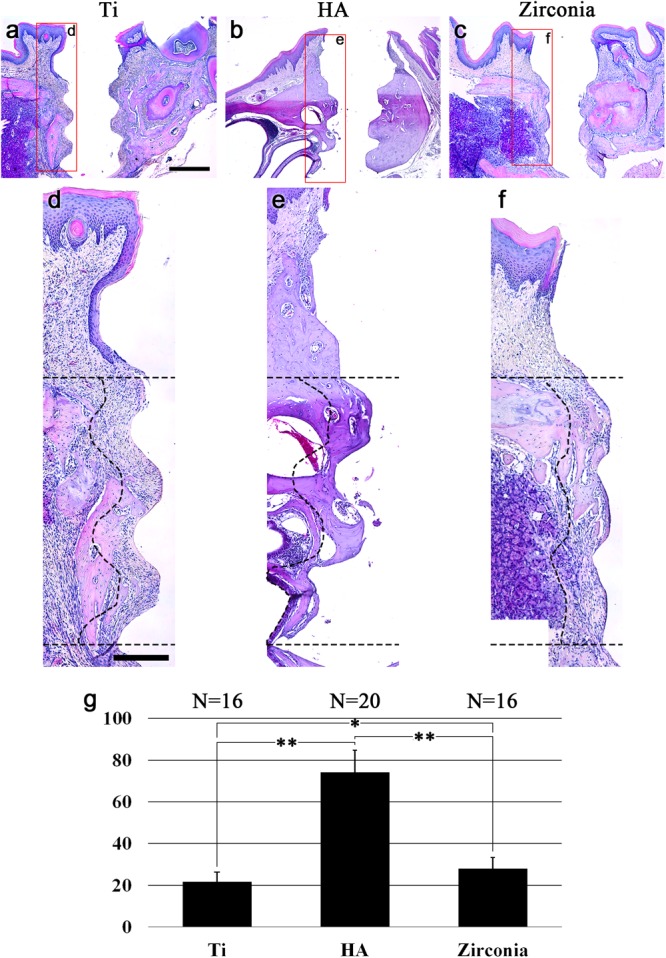
Bone healing after fixture transplantation. HE-stained images 8 weeks after transplantation of **(a)** noncoated titanium, **(b)** HA-coated titanium, and **(c)** zirconia implant fixtures. **(d–f)** High magnification of **(a–c)**. Dotted lines indicate the boundaries of the ROI for the bone area around the fixtures. The ROI is set from the top of the thread part of the fixture to the third thread crest and 150 μm from the surface of the fixture. **(g)** Bone ratio around the fixture. Bone healing was completed around HA fixtures 8 weeks after transplantation. Eight weeks are not enough for Ti and zirconia fixture bone healing. The results are expressed as the mean ± SD. ^∗^*p* < 0.05, ^∗∗^*p* < 0.005. Scale bars **(a–c)**, 500 μm; **(d–f)**, 200 μm.

Four transplanted Ti fixture samples and four zirconia fixture samples were harvested from each of the five transplanted mice (one fell off, respectively). All five HA fixtures were harvested without dislocation. Hematoxylin and eosin (HE) staining showed bone healing between the alveolar bone and the fixture (Figure 2). The alveolar bone was fully healed and showed osseointegration between the HA fixture and the alveolar bone at 8 weeks after transplantation (Figure 2b). However, alveolar bone healing on Ti and zirconia surfaces was not completed (Figure 2a,c) at 8 weeks. The osseointegration was partially found on the surfaces of the HA and zirconia implants, and the amount of healed bone was smaller than in the HA fixture (Figure 2d–f). The amount of bone around the fixture was measured. The bone-occupied area around the HA fixture had a mean value of 74.2%. The bone-occupied area of the Ti and zirconia fixtures were 21.7 and 28.0%, respectively (Figure 2g).

### Mucosal Seal on the Fixture

**FIGURE 3:**
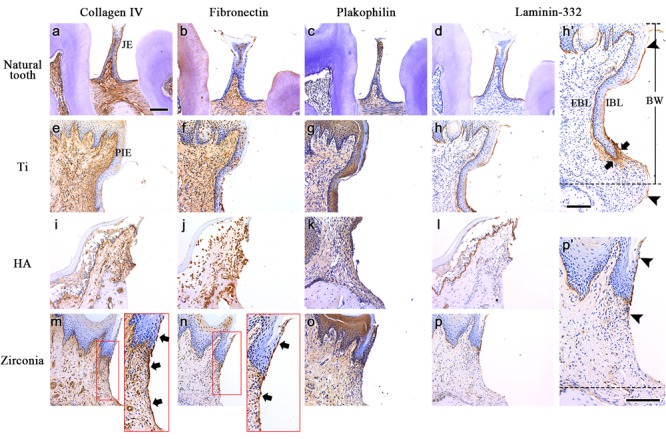
The expression of adhesive ECM molecules after the *in vivo* transplantation. IHC against collagen IV, fibronectin, plakophilin and laminin-332 was performed on natural tooth **(a–d)**, Ti **(e–h)**, HA **(i–l)**, and Zirconia fixtures **(m–p)**. The Arrows in the panel indicate continuous expression at the tooth contact surface of the PIE and connective tissue. **(h’,p’)** Extended images of panels h and p. Arrowheads indicate both ends of laminin-332 expression at the mucosa contact surface of Ti or zirconia fixtures (IBL). Connective tissue around apical leading edge of PIE shows strong expression of laminin-332 in Ti sample (arrows). Dotted lines indicate the alveolar crest levels of the healing bone. JE, Junctional epithelium; PIE, Peri-implant epithelium; IBL, Internal basal lamina; EBL, External basal lamina; BW, Biological width; All scale bars, 200 μm.

To determine how the mucosal seal forms depending on the fixture type, the expression of adhesive extracellular matrix (ECM) molecules after *in vivo* transplantation was analyzed. IHC against collagen IV, fibronectin, plakophilin and laminin-332 was performed. In the natural tooth, collagen IV was strongly expressed both at the tooth contact surface of the junctional epithelium (JE), which is called the internal basal lamina (IBL), and the ECM of connective tissue cells under the JE (Figure 3a). Fibronectin was expressed at the connective tissue and a few suprabasal cells of the JE (Figure 3b). Fibronectin was not expressed along the tooth and the JE interface (IBL). Plakophilin was expressed at the IBL and in the suprabasal cells. A weak expression in the external basal lamina (EBL, interface of JE and connective tissue) and connective tissue was observed (Figure 3c). Laminin-332 was strongly expressed at the IBL and weakly expressed at the EBL (Figure 3d). The connective tissue of the natural gingiva is mostly composed of gingival fibers, and the four kinds of ECM molecules were not expressed along the interface of the tooth and connective tissue (Figure 3a–d).

Unlike in the natural tooth, strong collagen IV expression was not observed along the contact surface of the PIE in the three types of fixtures (Figure 3e,i,m). It was expressed weakly only on the PIE of the zirconia implant. Collagen IV was expressed in the connective tissue of three type of fixtures as well as the natural gingiva. A unique feature is that collagen IV was expressed along the interface of the connective tissue and fixture continuously (Figure 3m, arrow).

The expression of fibronectin around the Ti and HA implant fixtures was similar to that of the natural tooth (Figure 3f,j). Some suprabasal cells of the PIE and connective tissue expressed fibronectin. In the zirconia implant, fibronectin was expressed in the connective tissue, similarly, to in the Ti and HA implants. And fibronectin was observed at the interface of the PIE (also called the IBL) and the fixture and at part of the continuous connective tissue and fixture interface (Figure 3n, arrows). Plakophilin expression was the same as that of the JE and natural gingiva connective tissue, suprabasal cells and connective tissues (Figure 3g,k,o).

Unlike other ECM molecules, laminin-332 was expressed along the implant fixture and PIE interface (IBL), similarly, as on the JE of the natural tooth (Figure 3h,i,p). The EBL and continuous basement membrane (BM) of sulcular and oral epithelium were also an expression area of the peri-implant gingiva. In the connective tissue, laminin-332 was not expressed, similarly, to the natural gingival connective tissue except Ti sample. Laminin-332 expression at the interface of the connective tissue and fixture was different depending on the fixture type. At the Ti fixture surface, laminin-332 was expressed along the IBL and continued to the interface of the connective tissue beneath the alveolar crest level of the healing bone (Figure 3h’ dotted line). Laminin-332 was strongly expressed in connective tissue around apical leading edge of PIE (Figure 3h’ arrows). However, Laminin-332 expression at the IBL of the zirconia fixture was limited to the end of the PIE (Figure 3p’). It did not continue to the connective tissue. The arrowheads indicate the initiation and the termination of the interface expression of laminin-332.

### Peri-Implant Epithelium Elongation

The length of the PIE of healed mucosa varied according to the type of fixture. The PIE elongation was checked on the mesial and distal sides of transplanted fixtures. PIE elongation was defined when the PIE made contact with the fixture and grew along the fixture surface more than 350 μm from the top. PIE elongation on the Ti fixture was observed at 14 sites in 16 mesial and distal sites of four mice. Figure 2d shows the longest PIE elongation among 14 positive sites of 4 mice, and it was 660 μm. The PIE elongation was found at 2 sites among 20 sites of five HA implanted mice. In the zirconia implanted cases, it was 2 sites among 16 sites of 4 mice.

**FIGURE 4:**
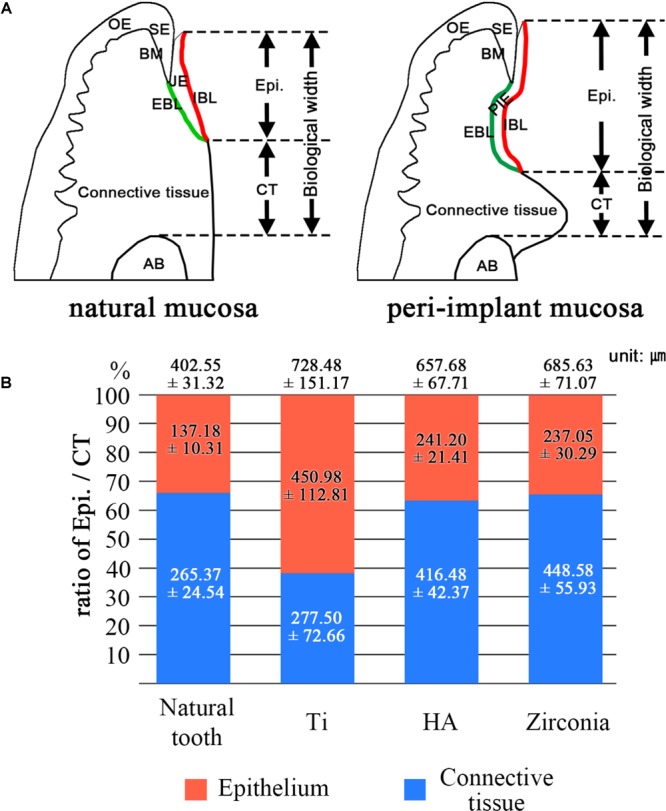
Composition of biological width. **(A)** Schematic images of natural mucosa and peri-implant mucosa. These show how to measure the components of the biological width. JE, Junctional epithelium; SE, Sulcular epithelium; OE, Oral epithelium; PIE, Peri-implant epithelium; IBL, Internal basal lamina; EBL, External basal lamina; BM, Basement membrane; AB, Alveoloar bone. **(B)** The ratio of epithelium and connective tissue was analyzed. The numbers on the graph mean the biological width. The biological width is composed of the epithelium and connective tissue lengths, which contact the tooth or implant fixture. The values were expressed as the mean ± SD. The ratio differences between the Ti group and the other groups were significant (*p* < 0.005).

The composition of the biological width (BW) was analyzed. From the top of the JE or PIE to the alveolar crest level, the heights of epithelium and connective tissue were measured (Figure 4A). The ratios between the epithelium and connective tissue are shown in Figure 4B. The contents of connective tissues were 65.9% in natural tooth, 38.1% in the Ti fixture, 63.3% in the HA fixture and 65.4% in the zirconia fixture. The ratio difference between the Ti fixture and the other groups was significant (*p* < 0.005). The ratio difference among the other groups except the Ti fixture was not significant. The mean BWs measured 402.55 μm in the natural tooth, 728.48 μm in Ti, 657.68 μm in HA, and 685.63 μm in zirconia.

## Discussion

The zirconia implant has been introduced as an alternative to the titanium implant due to several advantages (Ozkurt and Kazazoglu, 2011). Biomaterial studies for zirconia implants have accumulated considerably, while *in vivo* studies had not been performed yet. The present study is the first *in vivo* investigation using a small zirconia implant fixture in the mouse oral cavity. Dental implants require a mucosal seal to inhibit epithelial elongation and bacterial invasion of subepithelial connective tissues and implant interfaces (Pae et al., 2009). We analyzed the differences among machined surface Ti, HA-coated Ti and machined surface zirconia implant fixtures, which were transplanted in a similar way to how they are clinically applied to humans.

We observed how the proliferation of periodontal cells in each material occurred. Since the materials used are opaque, it is impossible to determine the features of the cells by optical methods. Thus, the WST-8 assay, which is able to count the number of cells indirectly through the culture media, was used. The WST-8 assay has been shown to be as effective as well-known colorimetric cell viability assays, such as MTT, XTT, and MTS assays (Tominaga et al., 1999). For analysis, periodontal tissue cells such as human cementoblast, human PDL fibroblast, and human gingival fibroblast were used. These cells were originated from mesenchymal dental follicle tissue. This contrasts with the gingival epithelium originated from the ectoderm. The material conditions were set to machined surface Ti, HA-coated Ti, and machined surface zirconia. All the three types of cells proliferated best on zirconia surfaces. The proliferation rates of cementoblasts were higher in the order of zirconia > Ti = HA. The PDL fibroblasts were in the order of zirconia > Ti > HA. The gingival fibroblasts were in the order of zirconia > HA > Ti. It was found that periodontal cells generally proliferate more prominently on zirconia surfaces. These results are comparable to previous studies. One study reported that cell properties on materials could be different depending on material type and roughness (Rutkunas et al., 2015). This study reported that cell proliferation among various properties was independent to material type and roughness. Another study showed zirconia is favored in terms of cell proliferation to titanium (Depprich et al., 2008). Considering the controversy of cell proliferation according to the material, the results of the present study may have a significant meaning.

Expression of ECM molecules is an important factor in adhesion to fixtures as well as proliferation of connective tissue in mucosal seals. Several previous studies have reported the expression of adhesion molecules on various implant materials. They showed zirconia can increase the adhesion capacity and the cellular growth rate of fibroblasts (Abrahamsson et al., 1998; Raffaelli et al., 2008; Okabe et al., 2016).

To mimick dental implants for clinical use in mouse oral cavities, the fixtures were manufactured as small as mouse maxillary first molars. This implant fixture was introduced in a previous study (Lee et al., 2017), and the zirconia fixtures were made with the same gauge. First, we examined the bone healing aspects of zirconia, Ti, and HA implants. Eight weeks was set as the healing period after transplantation because it took 8 weeks to achieve sufficient bone healing and osseointegration in the previous study using HA fixtures.

In the present study, bone healing of HA fixture was completed, while the bone healing of zirconia and Ti was not completed within 8 weeks. At the initial bone healing rate, HA fixture was the best among the three types of fixtures. It is well known that HA-coating on the implant surface induces rapid bone healing and shortens the time to initial loading (Kieswetter et al., 1996; Chaushu et al., 2001). The dislocation of one Ti and one zirconia implant in each group is judged to be due to the failure of initial stability following rapid bone healing. The bone healing rate between zirconia and Ti fixtures also showed differences through the histological analysis of bone structure around the fixture. The zirconia fixtures had significantly higher rate than Ti fixtures (*p* = 0.048). However, this difference between zirconia and Ti fixtures is not likely to be clinically meaningful.

The mucosal seal between the implant and adjacent mucosa is closely related to the initiation of peri-implantitis (Listgarten et al., 1991; Buser et al., 1992). The distributions of collagen type IV, laminin, fibronectin in the ECM and hemidesmosome were known as the critical factors in a healthy peri-implant mucosa interface (Silva et al., 2014; Araujo and Lindhe, 2018; Koidou et al., 2018). The tooth enamel surface contacts the JE of the mucosa and the tooth root contacts the connective tissue continuously. The adhesion of the JE to the enamel surface is achieved by several adhesion ECM molecules. The connective tissue of the natural gingiva is mostly composed of gingival fibers, which is dense connective tissue. The interproximal region is composed of trans-septal fibers linked to the tooth root surface directly. In other words, the connective tissue of the natural gingiva does not require a large amount of adhesion molecules at the surface where it contacts the tooth root. However, peri-implant connective tissue consists of loose connective tissues in which no fibers are directly linked to the implant surface. Therefore, some adhesion molecules are needed to form the mucosal seal of the connective tissue and implant interface.

In the present study, collagen IV was observed at the interface of the connective tissue and the three types of fixtures. It was observed at the zirconia-PIE interface as well as the tooth-JE interface, but not at the Ti- and HA-PIE interfaces. Among the three types, collagen IV was continuously expressed both of IBL and connective tissue-fixture interface only in zirconia. Fibronectin was observed at connective tissue interface and suprabasal cells of PIE of Ti and HA fixture samples. In zirconia implants, similarly to Collagen IV, fibronectin was expressed in IBL and connective tissue interface continuously. In contrast, plakophilin, which forms hemidesmosomes, showed a similar expression pattern to a natural tooth in all three types of implant fixtures. The expression pattern of collagen IV and fibronectin in the zirconia fixture, not observed in other fixtures, suggests a stronger mucosal seal formation at zirconia implant than other Ti and HA fixtures.

Laminin-332 was expressed in all IBL and EBL of JE and PIE. The expression at the interface of the connective tissue and fixture was observed to be different between the Ti and zirconia fixtures. The laminin-332 was expressed along the IBL and continued to the interface of the connective tissue and Ti fixture. The laminin-332 was also expressed strongly in the connective tissue around apical leading edge of PIE. However, it was limited to the IBL in zirconia fixtures. A previous study reported that laminin-332 induces cell migration during PIE formation after Ti implant transplantation in Rat (Atsuta et al., 2005a,b). These previous studies also showed that the leading edge of mucosal wound of oral sulcular epithelium and the underlying connective tissue express laminin-332 during oral mucosa wound healing. Considering the previous studies, it could be hypothesized that limitation of laminin-332 within PIE of zirconia regulate the elongation of PIE at the zirconia implant. However, the biological rationale of the relationship between laminin-332 and mucosal epithelium migration should be elucidated in further studies.

Peri-implant tissues are quite similar except for the PDL, although the nomenclature is slightly different. Therefore, the concepts of the healing process used in many periodontics can be applied to implant healing (Atsuta et al., 2016). One of them is the biological width (BW). The BW is defined as the JE (or PIE) length + connective tissue attachment length (same as from the JE-connective tissue interface to the alveolar bone crest level). In healthy gingiva, the BW is maintained at approximately 2.04 mm. A normal BW of peri-implant gingiva is considered to be 3.0–4.0 mm. In mice, the normal BW has not been determined yet. In the present study, the mean BWs were 402.55 μm in the natural tooth, 728.48 μm in Ti, 657.68 μm in HA, and 685.63 μm in zirconia implants, and they were located in the normal range proportionally.

The PIE serves as a primary physical barrier, but an active immune response to pathogens cannot be achieved through the PIE. Moreover, because PIE has a weaker adhesion than JE, a sufficient connective tissue site is required where blood vessels are abundantly distributed for effective protection. Therefore, the ratio of PIE in the BW should be properly maintained. In clinics, the guided tissue regeneration (GTR) procedure is used to prevent excessive migration of PIE (Murphy and Gunsolley, 2003).

The BW of the Ti fixture showed a significantly low connective tissue ratio. This is due to the elongation of the PIE. This phenomenon was not observed in HA, and bone healing was sufficient before PIE elongation occurred. The BW of zirconia fixture did not show the PIE elongation, although bone healing on zirconia was delayed compared to HA fixture. The results of the proliferation assay of periodontal cells, including HGF-1, may be indirect evidence of PIE elongation limitation in zirconia fixtures. It was concluded that rapid proliferation of connective tissue might affect to prevent PIE elongation.

Considering the regulation of PIE elongation by laminin-332 and the expression of adhesion molecules at the implant interface and connective tissue cell proliferation on materials, zirconia implants may tend to be more advantageous in terms of the mucosal seal than Ti implant. On the other hand, the bone contact area of the fixture appears to be advantageous for surface-treated titanium to achieve full osseointegration in a short time. To summarize these results, a new hybrid implant, a bone-contact thread portion composed of surface treated Ti alloy and a soft tissue-contact portion composed of zirconia, may have potential as a new alternative through further research.

## Data Availability

No datasets were generated or analyzed for this study.

## Ethics Statement

All the animal experiments were approved by the Yonsei University Health System Institutional Animal Care and Use Committee (YUHS-IACUC) in accordance with the Guide for the Care and Use of Laboratory Animals (National Research Council, United States). The animal study plan for these experiments (2016-0342) was reviewed and approved by this committee on January 28, 2017. All the experiments were performed in accordance with the guidelines of this committee.

## Author Contributions

D-JL, J-SR, and H-SJ conceptualized the study, designed the experiments. D-JL drafted the manuscript. D-JL and J-SR conducted the experiments. MS, K-WL, and J-ML critically revised the manuscript for intellectual content. D-JL, J-ML, and H-SJ analyzed and interpreted the results. All authors gave permission to the final draft of the manuscript.

## Conflict of Interest Statement

The authors declare that the research was conducted in the absence of any commercial or financial relationships that could be construed as a potential conflict of interest.
