# Supplementary material for: Differential Healing Patterns of Mucosal Seal on Zirconia and Titanium Implant
Source: Front Physiol. 2019 Jul 3;10:796. doi: 10.3389/fphys.2019.00796 (PMC6616312; doi:10.3389/fphys.2019.00796)
Supplement: Supplementary file 1 [file Table_1.DOCX]

**Differential healing patterns of mucosal seal on zirconia and titanium implant**

**
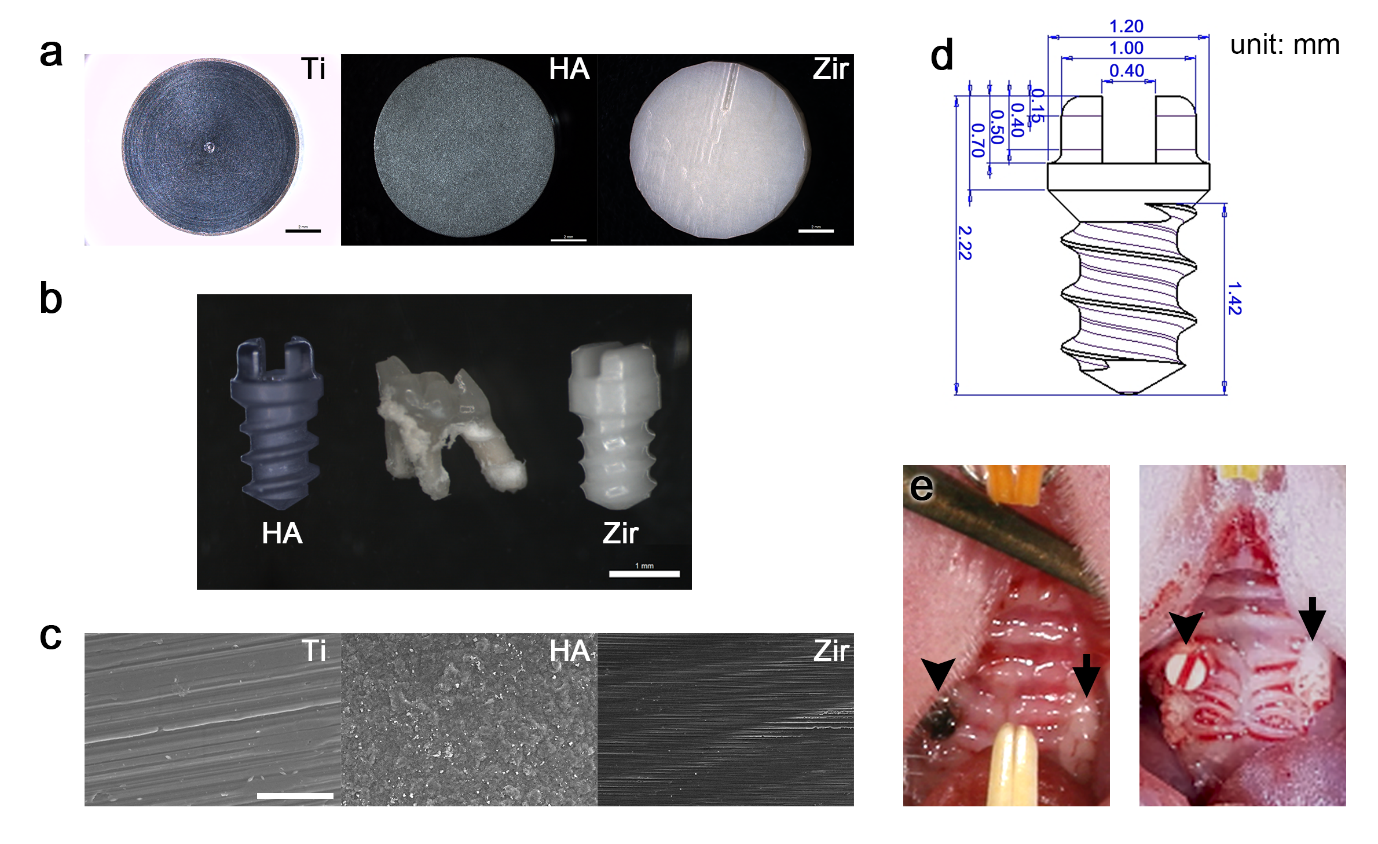
**

**Supplementary Figure 1 | Discs and Implant fixtures**

(a) Discs fabricated with Titanium (Ti), HA-coated titanium (HA), and zirconia (Zir). The diameters were 10.0 mm. scale bar, 2.0 mm. (b) Implant fixtures of HA and Zir. Fixtures are as small as mouse maxillary first molar. Scale bar, 1.0 mm. (c) Scanning electron microscope images of fixtures. Scale bar, 20 ㎛. (d) Blue print of fixture design. (e) Photographs of fixtures transplanted in the right maxillary first molar region (arroawheads) and natural first molar on the opposite side (arrows).
